# Case of a Luxation of the Thigh Bone

**Published:** 1784

**Authors:** 


VII. *Cafe of a Luxation of the Thigh Bone. Communicated to Dr.* Simmons *by Mr.* William Cribb, *Surgeon at Bifhop-Stortford, in Hertfordfhire.*

AS accounts of cafes in Surgery cannot be too frequently repeated, while there are any doubts of the poffibility of the accident, I will, with your permiffion, ftate the following,

ſing, in addition to the caſe publiſhed in the laſt Number * of the Medical Journal, of a compleat luxation of the femur. A boy, about nine years old, was the ſubject of this accident.—The head of the bone was ſituated in the foramen ovale, and the knee was drawn upwards and inwards by the ſtrong action of the flexor muſcles, which ſituation of the bone rendered the leg and thigh longer on the affected ſide than the other; and on bringing the knees together, the diſlocated bone protruded near three inches beyond its fellow. As I conſider a *fixed* counter extenſion as a matter never to be neglected in our attempts to reduce a luxated bone, I made my patient firm to a ſtaple at his head, and alſo to the table on which he was laid, by cloths paſſed between his thighs, and round his body. Mr. G. Cooper, Surgeon, of Haddam, who was ſo obliging as to aſſiſt me, made a ſteady extenſion in the direction in which the limb itſelf was drawn, while, with a napkin round the upper part of the thigh, I extended outwards and upwards, at the ſame time making a lever of the bone, by placing my hand on the knee; it was readily replaced, with a noiſe which was perceptible to every

* See page 312.

perſon person ptesent, and the boy immediately walked across the room, and in the course of a week was out at play when I called to see him.

As Wiseman, and other eminent Surgeons, have wholly denied the possibility of a luxation of the femur from the acetabulum, a relation of cases, which tend to overturn this erroneous hypothesis, become the more necessary, to put others on their guard, that they may not too readily subscribe to an opinion, which repeated facts have so often proved to be ill grounded.

*Bishop-Stortford,*

*Herts, December* 7, 1784.

